# Associations between Prenatal and Early Childhood Fish and Processed Food Intake, Conduct Problems, and Co-Occurring Difficulties

**DOI:** 10.1007/s10802-016-0224-y

**Published:** 2016-11-03

**Authors:** Maurissa SC Mesirow, Charlotte Cecil, Barbara Maughan, Edward D Barker

**Affiliations:** 1 Department of Psychology, Institute of Psychiatry, Psychology, and Neuroscience, King’s College London, 16 De Crespigny Park, London, SE5 8AF UK; 2 MRC Social, Genetic & Developmental Psychiatry Centre, Institute of Psychiatry, Psychology, and Neuroscience, King’s College London, London, UK

**Keywords:** Avon longitudinal study of parents and children (ALSPAC), Conduct problems, Strengths and difficulties questionnaire (SDQ), Fish, Processed food, Hyperactivity, Emotional difficulties

## Abstract

Little is known about early life diet as a risk factor for early-onset persistent conduct problems (EOP CP). To investigate this, we used data from the Avon Longitudinal Study of Parents and Children, a UK-based prospective epidemiological birth cohort. 5727 mother-child pairs (49.9 % boys) monitored since pregnancy (delivery date between 1 April, 1991 and 31 December, 1992) reported intake of fish and processed foods at 32 weeks gestation and, for the child, at 3 years; EOP (*n* = 666) and Low conduct problem (Low CP, *n* = 5061) trajectories were measured from 4 to 13 years; hyperactivity and emotional difficulties were assessed in childhood (4–10 years) and early adolescence (12–13 years), in addition to potential confounding factors (family adversity, birth complications, income). Compared to Low CP, mothers of EOP children consumed less fish (*p* < 0.01) and more processed food (*p* < 0.05) prenatally, while EOP children consumed more processed food at 3 years (*p* < 0.05). For EOP, but not Low CP children, consuming less than two servings/week of fish (vs. two or more servings/week, *p* < 0.05), and one or more servings/day of processed food (vs. less than one serving/day, *p* < 0.01), was associated with higher emotional difficulties in early adolescence. Conclusions: Findings suggest that prenatal and postnatal diets high in processed food, and low in fish, associate with an EOP CP trajectory and co-occurring difficulties in early adolescence. As small effect size differences were found, further studies are needed to investigate the long-term impact of early unhealthy diet.

**Electronic supplementary material:**

The online version of this article (doi:10.1007/s10802-016-0224-y) contains supplementary material, which is available to authorized users.

Early-onset persistent conduct problem (EOP CP) children are defined as having an onset of conduct problems (fighting, lying, stealing) before the age of 10 years, that persist through adolescence (Moffitt 2006). EOP CP is associated with host of co-occurring adjustment problems, including hyperactivity and emotional difficulties (Connor 2002). Risk factors for conduct problems (CP) include poverty, harsh parenting, maternal depression and anxiety, as well as poor care-giving environments (Barker and Maughan 2009). A risk factor that has received less attention, yet is likely important in early development, is the nutritional environment (Moffitt 2006). From pregnancy through the first 5 years of life, optimal nutrition is critical for normal child development in multiple domains of functioning (de Souza et al. 2011). Poor prenatal and early postnatal nutrition is not only associated with lower cognitive function (Barker et al. 2013), but also with higher conduct problems, emotional dysregulation, and hyperactivity (Liu and Raine 2011; Jacka et al. 2013). A focus on early nutrition is important, as diet is a targetable risk factor; improving maternal and/or childhood diet may help lower the prevalence of early-onset conduct problems, thus lowering substantial societal and economic costs associated with childhood CP and related adjustment problems (Hsia and Belfer 2008).

A healthy diet full of essential vitamins and minerals, including fatty acids, vitamin A, B-vitamins (e.g., folate, choline, thiamine), copper, zinc, iron, and selenium, is critical for proper neurological processes (Georgieff 2007). One particular nutrient that has been studied in recent years is omega-3 polyunsaturated fatty acids (PUFAs), primarily found in fatty fish and other seafood. Omega-3 PUFAs are essential for prenatal and early childhood brain growth and development (Innis 2009). Rapid accumulation of omega-3 PUFAs, such as docosahexaenoic acid (DHA) and eicosapentaenoic acid (EPA), starts in pregnancy, especially during the last trimester (Lauritzen et al. 2001). By 4 years of age, white matter tracts–bundles of myelinated axons that are high in omega-3 PUFAs–are already structured like adult brains (Hermoye et al. 2006). Insufficient amounts of dietary omega-3 s during this time frame can impede behavioural development via altered cell signalling and structure, neurotransmission (e.g., dopamine, serotonin), and cell membrane fluidity (Innis 2009; Simopoulos 2000). Such neurological impairments have been implicated in conduct disorder (Blair et al. 2006), hyperactivity, and emotional problems (Aoki et al. 2014).

Savoury processed foods, on the other hand, are abundant in vegetable and seed oils (e.g., soybean, corn, and sunflower oils), which have a high percentage of fat from omega-6 PUFAs compared to other healthy fats like fish oil. While some amount of omega-6 s are important for membrane structure and cell signalling pathways (Institute of Medicine 2005), consuming too much can interfere with neurological development through inhibition of omega-3 PUFA metabolism and availability (Novak et al. 2008). Longitudinal studies have revealed that prenatal and childhood intake of processed foods, which are high in vegetable and seed oils, are associated with childhood ADHD/conduct disorder (Jacka et al. 2013), hyperactivity (Wiles et al. 2007), and emotional difficulties (Kohlboeck et al. 2012).

Despite this research, the role of nutrition as a risk for early-onset persistent conduct problems is largely unknown. While there have been a handful of studies that have investigated the role of poor prenatal and childhood nutrition and later risk of emotional difficulties (Kohlboeck et al. 2012), dysregulated temperament (Pina-Camacho et al. 2015), hyperactivity (Howard et al. 2011), and conduct problems (Waylen et al. 2009), these studies have not focused on the early-onset and persistence of CP. The onset and persistence of conduct problems is important, as the earlier the onset, the greater the (a) co-morbidity of adjustment problems, such as emotional difficulties and hyperactivity, and the (b) risk for a life-course trajectory of antisocial behaviour and lifestyle (Moffitt 2006). Examining nutrition in relation to EOP and its co-occurring difficulties is important for understanding early life diet as a modifiable risk factor (Barker et al. 2013).

In the current study, we expanded upon previous research on diet-behaviour relationships to examine the impact of prenatal and 3y fish and processed food consumption on EOP children, and co-occurring difficulty risk. Based on previous studies, we had two main hypotheses: First, EOP children would be exposed to higher processed food and lower fish intake pre- and postnatal compared to Low CP children. Second, EOP children would show stronger associations between these dietary patterns and co-occurring difficulties (emotional difficulties, hyperactivity) compared to Low CP children. We also tested for potential sex differences as male foetuses may be more vulnerable to unhealthy nutrition in the last trimester due to more rapid biological growth during this developmental period (Mora et al. 1981).

## Method

### Sample

The Avon Longitudinal Study of Children and Parents (ALSPAC) is an ongoing study designed to understand the relationship between environmental and biological risk factors and health and development in children and parents. Eligible participants included all women residing in a defined area in southwest England with expected delivery dates between 1 April 1991 and 31 December 1992. The cohort was comprised of 14,541 initial pregnancies (13,988 children alive at 1 year), and has been followed for the past 23 years. The study sample was found to be representative of the general population of the United Kingdom at the start of data collection, except for a smaller proportion of mothers from ethnic minorities compared to the study location (4.1 % vs. 7.6 %), a higher proportion of married or cohabiting mothers, and a greater number of owner-occupier families (Boyd et al. 2013). All participants provided informed consent, and ethical approval for this study was obtained from ALSPAC Law and Ethics Committee and the Local Research Ethics Committee (http://www.bris.ac.uk/alspac/). The study website contains details of all available data through a fully searchable data dictionary (http://www.bris.ac.uk/alspac/researchers/data-access/data-dictionary/).

### Measures

#### SDQ: Conduct Problem Trajectories (4–13 years)

CP trajectories were created using the parent-reported SDQ conduct problem subscale. The SDQ is a widely used and validated psychometric measure of behaviour (Goodman 2001). From the 13,988 children alive at 1 year, only participants with at least four conduct problem data points available from 4-13y (4, 7, 8, 10, 12, 13 years) were selected for analyses. A series of growth models (one-group to six-group) were then fitted for the resulting 7218 children. A four-group developmental conduct problem trajectory was identified: early-onset persistent (EOP), childhood-limited (CL), adolescent-onset (AO), and “Low” conduct problems (Low CP) (Barker et al. 2010). From the four trajectories identified, only EOP (*n* = 666) and Low CP (*n* = 5061) trajectories were chosen for the current study (49.9 % boys). This was done to compare the most severe CP children (EOP) to typically developing children (Low CP), as AO and CL children have intermediate levels of CP scores.

The parent-reported SDQ conduct problem subscale consists of the following five items where parents reported on their child’s behaviour: “often has temper tantrums or hot tempers,” “generally obedient, usually does what adults request” [reverse coded], “often fights with other children or bullies them,” “often lies or cheats,” and “steals from home, school or elsewhere.” The five items are scored on a scale from 0 to 2 (0 = *not true*, 1 = *somewhat true*, 2 = *certainly true*). The scores for each of the five items were added together to get a total score out of 10 (0–3 = *normal*, 4 = *borderline*, 5–10 = *abnormal)*. These scores were then converted into binary indicators, with 0 = *not high risk* (normal + borderline), and 1 = *high risk* (abnormal) at each age based on established cut-off norms for 5–10-year-olds from England and Wales (Meltzer et al. 2000). These cut-offs have been shown to be strong predictors of conduct disorder (Goodman 2001).

#### SDQ: Co-Occurring Difficulties (4-13y)

Parent-reported SDQ subscales for emotional difficulties and hyperactivity were used as outcome measures for our second hypothesis. These two subscales were assessed by five items spanning 4–13 years. Children’s emotional difficulties were measured by the following five items: “Often complains of headaches, stomach-aches, or sickness,” “many worries, often seems worried,” “often unhappy, downhearted, or tearful,” “nervous or clingy in new situations, easily loses confidence,” and “many fears, easily scared.” Hyperactivity was measured by the following five items: “restless, overactive, cannot stay still for long,” “constantly fidgeting or squirming,” “easily distracted, concentration wanders” “thinks things out before acting,” [reverse coded] and “sees tasks through to the end, good attention span” [reverse coded]. For each subscale, all items across all ages were scored on a scale of 0–2 and summed together for a score ranging from 0 to 10, as described previously for conduct problem subscale.

Confirmatory Factor Analysis was then used to create childhood (4–10 years) and early adolescent (12–13 years) scores of emotional difficulties and hyperactivity. First order factors for emotional difficulties and hyperactivity scales were made to span two time periods: childhood (4–10 years) and early adolescence (12–13 years). Emotional difficulties showed acceptable fit to the data (factor loadings: *4–10 years:* 0.534–0.794; *12–13 years:* 0.778–0.780), as did hyperactivity (factor loadings: *4–10 years:* 0.638–0.867; *12–13 years:* 0.860–0.863). We used 10 years as a cut point in creating “childhood” and “early adolescence” measures to mirror the DSM-IV criteria for Conduct Disorder (≤10 years of age) and late-onset (after 10 years) (American Psychiatric Association 1994). This allowed us to compare EOP vs. Low CP for differences in co-occurring difficulties at these two stages of development.

#### FFQ: Fish and Processed Food (Pregnancy, 3y)

Dietary data were collected from the Food Frequency Questionnaire (FFQ), a valid and reliable measure of estimated food and beverage consumption (Rogers and Emmett 1998); The FFQ was used to assess (a) mother’s reported dietary patterns at 32 weeks gestation (“pregnancy”), and (b) what the mother reported feeding her child at 38 months of age (“3 years”). The FFQ contains questions about the consumption (portions per week) of various foods and beverages, with higher scores indicate higher frequency of consumption. Food records were used to give likely portion sizes during pregnancy and at 3 years, and standard portion sizes were adjusted to be age-appropriate (Emmett et al. 2002). Each of the FFQ items had five pre-determined responses: 1 = “never or rarely,” 2 = “once in 2 weeks,” 3 = “1–3 times per week,” 4 = “4–7 times per week,” and 5 = “more than once a day.” Based on a previous publication (Hibbeln et al. 2007), we converted scores into servings per week: 1 = *0 servings*; 2 = *0.5 serving*s; 3 = *2 servings*; 4 = *5.5 servings*; 5 = *10 servings*.

Continuous measures: To test the first hypothesis on between-group differences in fish and processed food consumption for CP trajectories, continuous variables were created from the following FFQ items. During pregnancy, three fish items (“white fish,” “oily fish,” and “shellfish”) and six processed food items (“fried foods,” “meat pies and pasties,” “chips,” “crisps,” “sausages or burgers,” and “pizza”) were used. At 3 years, three fish items (“white fish,” “other fish,” and “shellfish”), and eight processed food items (“pizza,” “sausages or burgers,” “meat pies and pasties,” “fried potatoes,” “chips,” “crisps,” “pot noodles,” and “fish fingers”) were used. Prenatal and 3y fish/seafood items were summed to create continuous prenatal and 3 years “fish” variables, respectively, describing weekly servings of fish consumed. The same procedure was repeated for prenatal and 3y savoury processed food items. The item “fish fingers” was included as a processed food because of its association with higher childhood fat-mass (Wosje et al. 2010).

Dichotomized measures: For the second hypothesis, continuous fish and processed food variables were dichotomized (according to dietary recommendations) to investigate interactions between food intake and CP trajectories, and associations with co-occurring difficulty outcomes. Fish was dichotomized as the following: less than two servings per week (“<2 servings/week”), and two or more servings per week (“≥2 servings/week”). The use of two servings per week as the cut point was based upon national dietary recommendations for pregnant women and children to consume two or more servings of fish and seafood per week (Scientific Advisory Committee on Nutrition 2004). Processed food was dichotomized into the following two groups: less than 1 serving per day (“<1 serving/day”), and 1 or more servings per day (“≥1 servings/day”), in line with dietary recommendations to reduce the amount of energy-dense, nutrient-poor foods, such as savoury processed foods (Gidding et al. 2005). Of note, grouping by weekly servings (as opposed to daily servings) could not be calculated due to the low number of EOP children (*n* = 4) who consumed less than one servings of processed food per week.

#### Covariates

Questions from ALSPAC were used to construct covariates for prenatal, birth, and childhood risks. Single measures of prenatal (18–32 weeks) and birth risk factors were dichotomized and scored as 0 (*without risk*) or 1 (*with risk*). Prenatal and birth risks included the following: one or more birth complications (abruption, cervical suture, preterm rupture); preterm birth (<37 weeks gestation); low birth weight (<2500 g at birth); never breastfed during first 6 months postnatal; multiparity; and any smoking during pregnancy.

The items from the Family Adversity Index (FAI) were also controlled for in our analyses. The FAI was developed by ALSPAC, and consists of risk factors that have been shown to relate to later child emotional and behavioural issues (Barker et al. 2012). These measures consist of 14 items, spanning pregnancy, 0–2 years postnatal, and 2–4 years postnatal. For each time period, the following factors were scored as 1 (with risk) or 0 (without risk): (1) early parenthood (first pregnancy <17 years or first birth <20 years); (2) inadequate housing (crowding, homelessness); (3) inadequate basic living conditions (no working bath/shower, no hot water, no indoor toilet, and/or no working kitchen); (4) housing defects (mould, roof leaks, pests [rats, mice, cockroaches]); (5) low educational attainment (unfinished compulsory schooling); (6) financial difficulties (poverty [social classes IV and V]); (7) no partner (not cohabiting, not in a relationship); (8) lack of partner affection (partner aggression, lack of intimacy); (9) partner cruelty (emotional and/or physical abuse from partner); (10) major family problems (taken into care, not with mother); (11) large family size (three or more children); (12) maternal psychopathology (anxiety, depression [including postnatal], attempted suicide); (13) substance use (hard drugs, alcoholism, or alcohol consumption ≥2 drinks per day); and (14) criminal history (involvement with police, criminal convictions).

All prenatal, birth, and FAI risk factors were all summated into an index and regressed on all study variables. Further computational explanations are described elsewhere (Barker et al. 2013). Consistent with previous analyses (Kohlboeck et al. 2012) FFQ-derived prenatal and 3y total daily energy (kJ) intake were also used as covariates.

### Attrition and Missing Data

Completed data on prenatal fish and processed food consumption was available for 95.9 % (*n* = 5493) of the subsample. At 3y, complete information for both fish and processed food consumption was available for 95.6 % (*n* = 5473) and 95.7 % (*n* = 5480) of the subsample, respectively. Childhood SDQ scores (hyperactivity, emotional difficulties) were available for 92.0 % of EOP (*n* = 614) and 93.6 % of Low CP (*n* = 4737) children. Adolescent SDQ scores were available for 85.7 % of EOP (*n* = 571) and 88.8 % of Low CP (*n* = 4493) children.

Barker and Maughan (2009) previously tested all risk factors for conduct problems as predictors of exclusion for the sample of 7218 children. Mothers who were excluded from the original analyses reported higher levels of sociodemographic risks (early motherhood: odds ratio [OR] = 1.42; no maternal basic education (OR = 1.98) or mid-level (OR = 1.50) qualifications; maternal smoking during pregnancy (OR = 1.64)). We further tested these risk factors as predictors for exclusion for the current sample of 5727 children (EOP and Low CP). Mothers excluded from the current analyses did not differ in risk factors from the original 7218 included in the total set of CP trajectories. The excluded sample did not differ from the 5727 children in fish consumption, but reported higher levels of processed food than Low CP, but lower levels than the EOP group (*p* < 0.01). The excluded children also differed in emotional difficulties (*p* < 0.001) and hyperactivity (*p* < 0.001) SDQ scores, having higher scores than the Low CP group, but lower scores than the EOP group.

### Statistical Analysis

Analyses were performed in SPSS version 22, in two steps. In the first step, a 2 (EOP vs. Low CP) × 2 (boys vs. girls) univariate ANOVA factorial design was performed using ordinary least squares. Mean weekly portions and multivariate F-values of fish and processed food consumption during pregnancy and 3y were generated for EOP and Low CP boys and girls. All estimates control for total energy intake (prenatal, 3 years) and risk factors. Effect sizes were calculated for main effects (partial eta squared [*η*_*p*_^*2*^]), and between-group contrasts (Cohen’s *d,* Hedge’s *G*).

In the second step, 2 (EOP vs. Low CP) × 2 (fish [“<2 servings/week” vs. “≥2 servings/week”], or processed food [“<1 serving/day” vs. “≥1 servings/day]”) univariate ANOVA tests were performed to test associations between these foods and SDQ emotional difficulties and hyperactivity in childhood (4–10 years) and early adolescence (12–13 years) by CP trajectory, controlling for sex. After performing the 2 × 2 ANOVAs, one-way ANOVAs were used to compare number of servings of fish (<2 servings/week vs. ≥2 servings/week) and processed food (<1 serving/day vs. ≥1 servings/day) on co-occurring difficulty outcomes in childhood and early adolescence. These one-way ANOVAs were performed on EOP and Low CP trajectories individually. Effect sizes were calculated for main effects (partial eta squared [*η*_*p*_^*2*^]) and for between-group contrasts (Cohen’s *d*, Hedge’s *G*).

## Results

### Sample Characteristics

Study characteristics for prenatal and childhood risk factors, and summary statistics for child co-occurring difficulties, by CP group are shown in Table 1. Overall, mothers of EOP children were more likely to come from low-SES backgrounds (*p* < 0.001), smoke during pregnancy (*p* < 0.001), and never breastfeed during the first six months postnatal (*p* < 0.01). In addition, EOP children were more likely to be exposed to a variety of contextual risk factors (e.g. poor housing conditions, financial difficulties, maternal psychopathologies and substance abuse) prenatally through the first four years of life (*p* < 0.01).

1. Fish and Processed Food Consumption by CP Trajectory

**Table 1:** Maternal and child sample characteristics

	EOP		Low CP		Difference ^a^
Maternal and birth characteristics	%	n/N	%	n/N	Χ^2^
Ethnicity (Caucasian/white)	98.1	635 / 647	98.6	4891 / 4961	0.78
Offspring sex (male)	56.9	379 / 666	49.0	2478 / 5061	14.86***
Multiparity	67.3	439 / 652	64.0	3194 / 4993	2.84
Social class: low-SES ^b^	14.3	73 / 525	8.4	364 / 4344	17.50***
Prenatal and birth risk factors					
Ever smoked during pregnancy	30.9	203 / 656	16.0	799 / 5001	89.09***
Preterm (<37 weeks gestation) ^c^	3.60	24 / 666	4.35	220 / 5061	0.80
Low birth weight (<2500 g) ^c^	3.20	21 / 656	3.46	173 / 5001	0.12
Presence of birth complications	22.7	136 / 599	19.6	903 / 4618	3.30
Never breastfed (first 6 months)	20.1	133 / 529	15.3	771 / 5039	10.06**
Early parenthood ^d^	7.4	49 / 666	3.6	184 / 5061	20.89***
Contextual risk factors (prenatal-4 years)					
Inadequate Housing	21.5	143 / 666	10.9	553 / 5060	61.26***
Lack of basic living conditions	9.3	62 / 666	7.1	360 / 5057	4.18*
Housing defects/infestations	32.2	214 / 666	26.0	1313 / 5059	11.65**
No educational qualifications ^e^	18.1	117 / 648	9.0	451 / 4988	51.42***
Financial difficulties	30.3	201 / 664	17.4	877 / 5054	64.02***
Partner status (no partner)	20.0	133 / 665	13.2	667 / 5060	22.73***
Lack of partner affection	40.6	263 / 647	17.8	891 / 4995	183.20***
Partner cruelty	38.0	253 / 666	17.7	894 / 5059	151.63***
Major family problems	3.3	22 / 666	3.0	150 / 5061	0.22
Large family size (>3 children)	9.2	61 / 665	6.4	322 / 5061	7.45**
Maternal psychopathology	55.7	371 / 666	28.4	1437 / 5061	203.22***
Substance use ^e, f^	26.9	179 / 666	20.0	1010 / 5061	17.13***
Trouble with police ^e^	11.7	78 / 666	4.9	248 / 5059	50.82***
Criminal convictions ^e^	1.8	12 / 666	0.9	44 / 5059	5.28*
Child behavioural characteristics ^g^	mean ± SD	n/N	mean ± SD	n/N	F
Emotional difficulties					
Childhood (4-10y)	2.47 ± 1.56	666 / 666	1.28 ± 1.17	5061 / 5061	558.88***
Early Adolescence (12-13y)	2.63 ± 2.03	621 / 666	1.15 ± 1.34	4797 / 5061	587.72***
Hyperactivity					
Childhood (4-10y)	5.49 ± 2.13	666 / 666	2.81 ± 1.68	5061 / 5061	1402.50***
Early Adolescence (12-13y)	5.27 ± 2.38	620 / 666	2.27 ± 1.73	4797 / 5061	1491.33***

*EOP* early-onset persistent conduct problems, *Low CP* low conduct problems, *n* number with reported adversity, *N* total number with data available, *SD* standard deviation, *SES* socioeconomic status

*p < 0.05; **p < 0.01; ***p < 0.001

^a^df = 1 (Pearson”s chi-square; F-values)

^b^Classes IV and V (Registrar General”s social class scale, Surveys 1991)

^c^Word Health Organization

^d^Defined as first pregnancy <17 years or first birth <20 years

^e^mother or partner

^f^Includes alcohol, illicit drug use, and more than two alcoholic drinks/day

^g^Mean scores (parent-reported, 10-point scale), Strengths and Difficulties Questionnaire

**Fig. 1:**
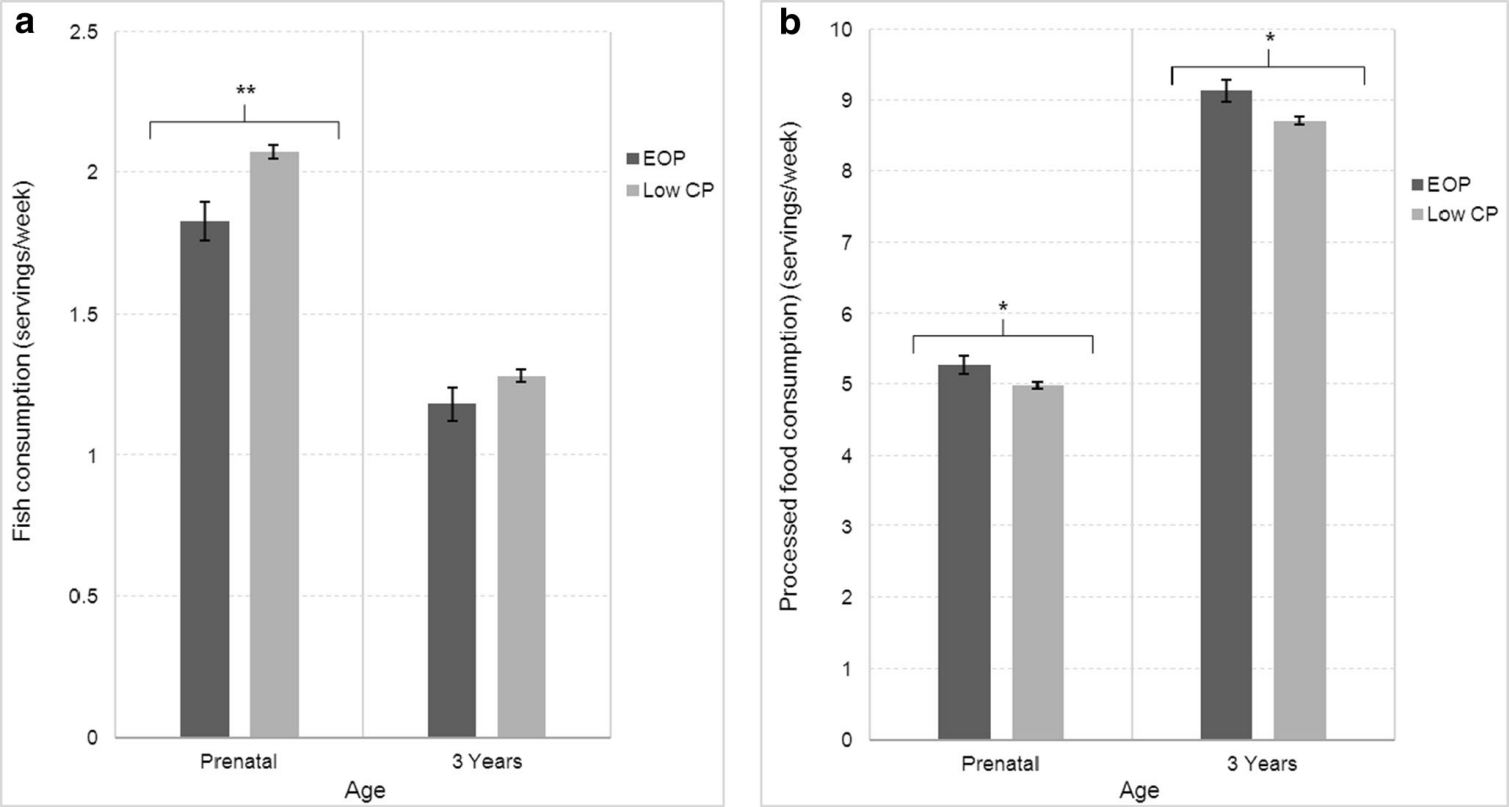
**a**-**b** Servings per week of **a** fish, and **b** processed food, at pregnancy and 3 years, by CP trajectory. **a** Servings/week of fish, for EOP (*n* = 616) and Low CP (*n* = 4732) children, prenatally (32 weeks gestation) and 3 years. **p < 0.01. *Note*. EOP = early-onset persistent conduct problems; Low CP = low conduct problems. **b** Servings/week of processed food, for EOP (*n* = 616) and Low CP (*n* = 4732) children, prenatally (32 weeks gestation) and 3 years. *p < 0.05. *Note*. EOP = early-onset persistent conduct problems; Low CP = low conduct problems

Mean weekly servings of fish and processed food for EOP and Low CP trajectories (prenatal, 3 years) along with overall group differences (F-statistics) are shown in Table 2. Group differences are given for main effects (CP trajectory [EOP vs. Low CP], sex) and the interaction (CP trajectory*sex). For EOP vs. Low CP, fish and processed food consumption differed both prenatally and at 3 years. Independent of sex, maternal prenatal fish consumption (servings/week) was lower for EOP mothers compared to Low CP (*p* = 0.001, Fig. 1a). EOP processed food consumption (servings/week) was higher in pregnancy and 3y compared to Low CP (*p* < 0.05, Fig. 1b). One sex difference was found; at 3 years, boys consumed less fish than girls, independent of CP trajectory. There were no significant interactions between CP trajectory and sex for fish or processed food at either time point. The overall effect size for the main effect of CP trajectory was small (*η*_*p*_^*2*^ = 0.001–0.002), as was the effect size for sex differences for fish consumption at 3 years (*η*_*p*_^*2*^ = 0.001). Between-group effect sizes for EOP vs. Low CP was small-to-medium (*G* = 0.18–0.25), and was small (*d* = 0.004–0.09) for boys vs. girls (Supplementary Table S1, located online).

STEP 2: Fish, processed food, and co-occurring difficulties

**Table 2:** Weekly servings of fish and savory processed food consumption at pregnancy and 3 years, by CP trajectory and sex

Food	Age	Mean weekly servings (SE)				F-values ^a^					
		EOP		Low CP		Conduct Problem Trajectory		Sex		Conduct Problem Trajectory*Sex	
		Boys	Girls	Boys	Girls	F	p	F	p	F	p
		(n = 348)	(n = 268)	(n = 2312)	(n = 2420)						
Fish	Prenatal	1.86 (0.09)	1.79 (0.10)	2.09 (0.03)	2.06 (0.03)	11.49	0.001 ^b^	0.43	0.51	0.12	0.73
	3 years	1.11 (0.08)	1.25 (0.09)	1.21 (0.03)	1.35 (0.03)	2.46	0.12	5.02	0.025 ^e^	<0.001	0.99
Processed Food	Prenatal	5.25 (0.17)	5.30 (0.19)	4.94 (0.07)	5.02 (0.06)	4.45	0.035 ^c^	0.24	0.63	0.02	0.90
	3 years	9.20 (0.20)	9.09 (0.23)	8.69 (0.08)	8.76 (0.08)	6.52	0.011 ^d^	0.02	0.89	0.27	0.60

(CP trajectory (EOP vs. Low CP]) × 2 (sex) univariate ANOVA comparing mean weekly servings of fish and process food in pregnancy and 3 years. Overall group differences (F-values) are given for main effects (CP trajectory, sex) and interaction (CP trajectory*sex). All estimates control for total energy intake (prenatal, 3 years) and contextual risk factors. EOP = early-onset persistent conduct problems; Low CP = low conduct problems. Effect sizes (partial eta squared): ^b^ *η*_*p*_^*2*^ = 0.002; ^c^ *η*_*p*_^*2*^ = 0.001; ^d^ *η*_*p*_^*2*^ = 0.001; ^e^ *η*_*p*_^*2*^ = 0.001

Mean SDQ scores for emotional difficulties and hyperactivity (childhood, early adolescence) and overall group differences are shown in Table 3. Mean SDQ scores were analysed with respect to low vs. high intakes of fish and processed food, and F-statistics were measures for overall group differences for the main effects (CP trajectory, daily/weekly servings) and interactions (CP trajectory*servings). Four main findings are highlighted here.

**Table 3:** SDQ scores childhood and adolescence, by conduct problem trajectory and fish processed food consumption

SDQ scores (by food type)	Mean SDQ scores (SE)				F-values ^a^		
	EOP		Low CP				
Fish (prenatal) ^b^	<2 svgs/wk	≥2 svgs/wk	<2 svgs/wk.	≥2 svgs/wk.	F_CP_^e^	F_Fish_	F_CP*Fish_
Emotional difficulties (4-10y)	0.58 (0.04)	0.54 (0.06)	-0.16 (0.02)	-0.17 (0.02)	358.87***	0.44	0.14
Emotional difficulties (12-13y)	0.62 (0.04)	0.44 (0.06)	-0.16 (0.02)	-0.19 (0.02)	359.32***	8.17** ^f^	4.94* ^i^
Hyperactivity (4-10y)	0.87 (0.04)	0.80 (0.06)	-0.29 (0.02)	-0.29 (0.02)	905.59***	1.01	0.82
Hyperactivity (12-13y)	0.90 (0.04)	0.88 (0.06)	-0.24 (0.02)	-0.26 (0.02)	934.49***	0.14	0.00
Processed food (prenatal,3y) ^c^	<1 svg/day	≥1 svg/day	<1 svg/day	≥1 svg/day	F_CP_^e^	F_Processed Food_	F_CP*Processed Food_
Emotional difficulties (4-10y)	0.52 (0.12)	0.58 (0.04)	-0.20 (0.04)	-0.17 (0.01)	125.85***	0.47	0.05
Emotional difficulties (12-13y)	0.20 (0.11)	0.58 (0.04)	-0.23 (0.04)	-0.17 (0.01)	87.85***	12.58*** ^g^	6.86** ^j^
Hyperactivity (4-10y)	0.69 (0.12)	0.86 (0.04)	-0.41 (0.04)	-0.28 (0.01)	310.43***	5.78* ^h^	0.06
Hyperactivity (12-13y)	0.79 (0.12)	0.91 (0.04)	-0.35 (0.04)	-0.24 (0.01)	326.01***	3.04	0.01

(CP trajectory [EOP CP vs. Low CP]) × 2(servings) univariate ANOVAs comparing standardized mean scores (z-scores) of emotional difficulty and hyperactivity by CP trajectory and servings of fish or processed food. Overall group differences (F-values) are given for main effects (CP trajectory, servings) and interaction (conduct problem trajectory*servings). All estimates control for total energy intake (prenatal, 3 years), sex, and contextual risk factors. *EOP* early-onset persistent conduct problems, *Low CP* low conduct problems; 4-10y = 4–10 years; 12-13y = 12–13 years

^a^df = 1

^b^“<2 servings/week” vs. “≥2 servings/week”

^c^“<1 servings/day” vs. “≥1 servings/day”

Effect sizes (partial eta squared): ^e^ *η*_*p*_^*2*^ = 0.019–0.162; ^f^ *η*_*p*_^*2*^ = 0.002; ^g^ *η*_*p*_^*2*^ = 0.003; ^h^ *η*_*p*_^*2*^ = 0.001; ^i^ *η*_*p*_^*2*^ = 0.001; ^j^ *η*_*p*_^*2*^ = 0.002

First, as would be expected, EOP children had higher emotional difficulty and hyperactivity scores in childhood (*p* < 0.001) and early adolescence (*p* < 0.001) compared to Low CP children. The effect size for the main effect of CP trajectory was small to medium (*η*_*p*_^*2*^ = 0.02–0.16), reflecting differences in emotional difficulties and hyperactivity between EOP and Low CP children during childhood and early adolescence (Supplementary Table S2, located online).

Second, the main effect of processed food consumption, independent of CP trajectory, was associated with higher hyperactivity at 4–10 years (*p* < 0.05). The main effect of processed food on hyperactivity had an overall small effect size (*η*_*p*_^*2*^ = 0.001), with a moderate between-group effect size for EOP and Low CP trajectories (*G* = 0.23).

**Fig. 2:**
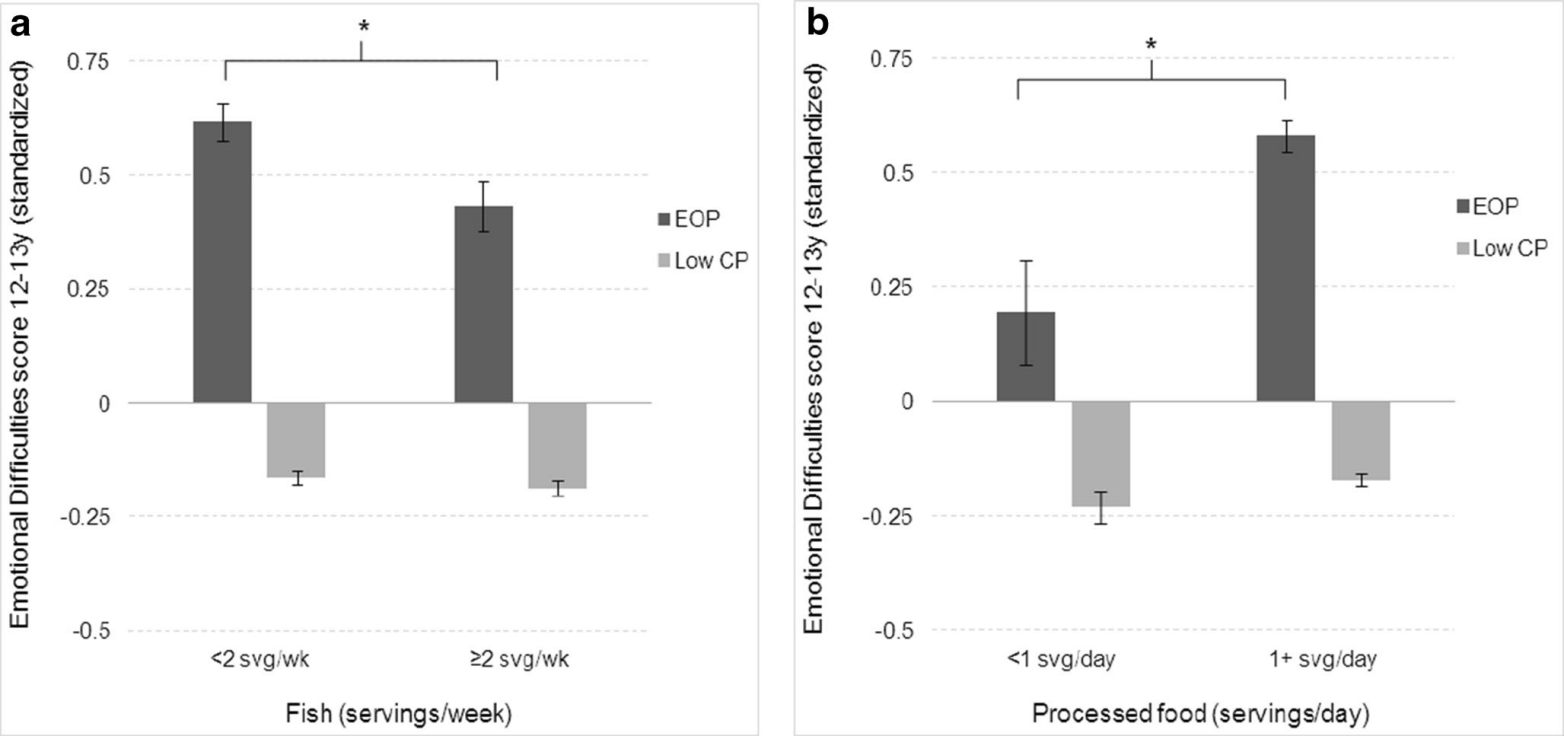
**a**-**b** Consumption of **a** fish, and **b** processed foods, and adolescent emotional difficulties, by CP trajectory. **a** Emotional difficulties scores (12–13 years) and prenatal fish consumption, by CP trajectory. *Note*. EOP = early-onset persistent conduct problems; Low CP = low conduct problems; “<2 svg/week”: less than 2 servings per week; “≥2 svg/wk”: 2 or more servings per week. *p < 0.05. **b** Emotional difficulties scores (12–13 years) and prenatal + 3y processed food consumption, by CP trajectory. *Note*. EOP = early-onset persistent conduct problems; Low CP = low conduct problems. “<1 svg/day”: less than 1 servings per day; “≥1 svg/day”: 1 or more servings per day. *p < 0.05

Third, there was an interaction between prenatal fish consumption and CP trajectory, and associations with early-adolescent emotional difficulties. The effect size for the overall interaction was small (*η*_*p*_^*2*^ = 0.001). When observing associations between fish and emotional difficulties for EOP only, prenatal consumption of less than two servings per week of fish was associated with higher early-adolescent emotional difficulties compared to those who consumed two or more servings per week (*p* < 0.01, Fig. 2a), with small overall (*η*_*p*_^*2*^ = 0.01) and between-group (*G* = 0.16, Supplementary Table S2) effect sizes. There were no significant differences between EOP boys and girls (*F* = 0.40, *p* = 0.53). There were no differences in early-adolescent emotional difficulties with respect to fish consumption for Low CP children (*G* = 0.03).

Fourth, there was an interaction between prenatal and 3y processed food consumption and CP trajectory, and associations with early-adolescent emotional difficulties. The effect size for interaction between CP trajectory and processed food was small (*η*_*p*_^*2*^ = 0.002). When observing associations between processed food and emotional difficulties for EOP only, consumption (prenatal, 3 years) of one or more servings per day of processed food was associated with higher early-adolescent emotional difficulties compared to those who consumed less than one servings per day (*p* < 0.01, Fig. 2b), with small overall (*η*_*p*_^*2*^ = 0.01) and medium between group (*G* = 0.38, Supplementary Table S2) effect sizes. There were no significant differences between EOP boys and girls (*F* = 2.24, *p* = 0.14). There were no differences in early-adolescent emotional difficulties with respect to processed food consumption for Low CP children (*G* = 0.09).

## Discussion

Using a UK population-based prospective study, we examined the relationship between early life fish and processed food intake, early onset conduct problem trajectories, and co-occurring difficulties. The results of this study inform existing research in three main ways.

Firstly, after controlling for risk exposures and total energy intake, maternal prenatal diet of EOP children was lower in fish and higher in processed food compared to intake for Low CP children, and EOP children consumed more processed food at 3 years. These findings are in line with previous studies that found adolescents with conduct problems or related difficulties experienced poor nutrition in early childhood ( Liu et al. 2004; Raine et al. 2003). To our knowledge, this is the first time that consumption of specific foods (i.e., fish, processed food) has been found among EOP children prenatally and in early childhood.

It is important to note that the current results differ from a similar study that also used ALSPAC data (Waylen et al. 2009), who found no difference in prenatal omega-3 intake (derived from FFQ fish consumption) for children diagnosed with or without ODD/CD at 8 years old. However, this may be due to the cohort selected. In the current study, we used data from longitudinal trajectory groupings of conduct problems between ages 4–13 years, rather than a diagnostic score for CD/ODD at one time point (8 years). This allowed us to investigate fish intake among a larger cohort of children who have a conduct problem trajectory that spans childhood and early adolescence (EOP, *n* = 666) compared to children in a low conduct problem trajectory. Waylen et al. (2009) chose to compare children who met clinical diagnosis for ODD/CD at 8 years to those who did not, which resulted in a smaller sample size (*n* = 189), and limiting analyses to children who might not be part of the EOP conduct problem trajectory, as an ODD/CD diagnosis at 8 years might not persist through early adolescence.

Secondly, after controlling for risk exposures, total energy intake, and sex, consuming at least two servings of fish every week during pregnancy was associated with lower emotional difficulties at 12–13 years for EOP, but not Low CP, children. Our findings expand on the knowledge of previous studies that found associations between higher birth DHA serum levels (Kohlboeck et al. 2011), and short-term use of omega-3 PUFA supplementation (Kirby et al. 2010), and lower childhood emotional difficulties and conduct problems. In the current study, however, we found effects in early adolescence (12–13 years) but not in childhood (4–10 years), which may be due to the role of foetal programming on later health outcomes. When nutritional insults occur during a critical developmental period (e.g., pregnancy), neurodevelopmental and behavioural changes can occur that can last into adulthood, and are not always observed in childhood (Bennet and Gunn 2006). In the Dutch Famine Study, undernutrition (not receiving enough essential nutrients) during the second and third trimesters was associated with affective disorders in the offspring when they were 18 years and older (Brown et al. 2000). In the present study, it may be that the adolescent period, with increased autonomy from parents and exposure to increasingly complex social interactions, represents an increased period of stress that can potentiate the early vulnerabilities established in part by poor early life nutrition. As the results of nutritional insults in early life might, under certain circumstance, manifest much later (e.g., in adolescence), our findings may reflect a delayed onset of emotional difficulties.

Thirdly, this study extends current knowledge in associations between processed foods and early-adolescent difficulties. Consuming at least one serving of savoury processed food every day in pregnancy and 3 years was associated with (a) higher childhood hyperactivity for both Low CP and EOP children, and (b) higher early-adolescent emotional difficulties among EOP children. Our findings expand upon previous research on associations between “junk food” (defined as high-fat/sugar foods) and behavioural difficulties among the ALSPAC cohort (Peacock et al. 2011; Wiles et al. 2007). While the previous studies did not find associations between junk food and emotional difficulties, our findings differ in three important ways: first, we focused on EOP CP children who already have a high risk of co-occurring difficulties (Connor 2002), rather than focusing on typically developing children. Second, we limited our analyses to savoury processed foods, as excluding sugary processed foods may help to reduce the possible confounding and contradictory effects of sugar on brain development (Beilharz et al. 2014; Maniam et al. 2015). Third, we investigated associations between prenatal and 3 year diet – time periods that are more susceptible to nutritional insults – and early-adolescent outcomes, rather than focusing on a less critical age range for junk food (4.5 years and 7 years). As the previous studies only focused on typically developing children, findings are limited in their application to high-risk groups such as EOP CP children, who are more likely to have co-occurring difficulties that persist into adolescence (Moffitt 2006).

Six main limitations must be taken into account when interpreting the findings of this study. Firstly, adults are likely to underreport intake of energy-rich, nutrient-poor foods in dietary assessments (Bingham 1994). As the FFQs were parent-reported, there may be an underestimation of overall intake of processed food in pregnancy and at 3y. Secondly the FFQ does not distinguish between oily fish and non-oily fish at 3 years, with prenatal “oily fish” being replaced with “other fish” on the questionnaire at 3 years. This lack of distinction may underestimate the effects of omega-3 rich oily fish on behavioural development. Thirdly, mothers of EOP children are more likely to face adversities, and so are less likely to have initiated or continued participation in the study (Wolke et al. 2009), meaning our findings may be conservative. Fourthly, there is a relatively low representation of ethnic minorities, despite a broad range of socioeconomic backgrounds. Future studies would need to be performed in more ethnically diverse settings. Fifthly, while the present study controlled for a wide range of prenatal and postnatal contextual risk factors, it did not investigate biological mechanisms (e.g., DNA methylation) that might explain associations between diet and co-occurring difficulties. Lastly, our effect sizes were small. Our findings should be interpreted with caution, and further replication will be needed.

## Conclusion

To the best of our knowledge, the present study is the first to examine specific healthy (fish) and unhealthy (processed) foods with respect to the risk for early-onset persistent conduct problems and co-occurring difficulties. With the use of a large prospective cohort, we found evidence of differences in fish and processed food consumption among mothers of EOP vs. Low CP children in pregnancy, and in children’s processed food consumption at 3 years. Moreover, lower prenatal fish consumption, and higher pre- and postnatal processed food consumption, were both associated with higher adolescent emotional difficulties. In the UK, children and adults consume below the current recommendations for weekly servings of fish, and above weekly recommendations for fats and processed foods (Bates et al. 2014). Further studies are needed to investigate the potential role of early-life diet and risk for conduct problems. Interventions that increase fish and reduce processed food consumption in early-life can help reduce risks of early-onset persistent conduct problems and co-occurring difficulties.

## Electronic supplementary material

ESM 1 (DOCX 11 kb)

ESM 2 (DOCX 13 kb)
