# Supplementary material for: Associations between Prenatal and Early Childhood Fish and Processed Food Intake, Conduct Problems, and Co-Occurring Difficulties
Source: J Abnorm Child Psychol. 2016 Nov 3;45(5):1039–49. doi: 10.1007/s10802-016-0224-y (PMC5415431; doi:10.1007/s10802-016-0224-y)
Supplement: Supplementary file 1 — (DOCX 11 kb) [file 10802_2016_224_MOESM1_ESM.docx]

Supplementary Table S1. Effect Sizes for Group Differences on FFQ scores, for step 1 (*Cohen’s d*)

| *FFQ* | EOP vs. Low CP ^a^ | Sex  (boys vs. girls) | EOP  (boys vs. girls) | Low CP  (boys vs. girls) |
| --- | --- | --- | --- | --- |
| *Prenatal* |  |  |  |  |
| Fish | 0.18* | 0.02 | 0.04 | 0.01 |
| Processed Food | 0.23** | 0.01 | 0.02 | 0.03 |
| *3 years* |  |  |  |  |
| Fish | 0.09 | 0.06 | 0.08 | 0.08 |
| Processed Food | 0.25** | 0.004 | 0.09 | 0.02 |

*Note*. EOP=early-onset persistent conduct problems; Low CP=low conduct problems; *small effect size, **medium effect size, ***large effect size.

^a^ Effect sizes measured as Hedge’s *G* due to difference in sample sizes
