# Supplementary material for: Associations between Prenatal and Early Childhood Fish and Processed Food Intake, Conduct Problems, and Co-Occurring Difficulties
Source: J Abnorm Child Psychol. 2016 Nov 3;45(5):1039–49. doi: 10.1007/s10802-016-0224-y (PMC5415431; doi:10.1007/s10802-016-0224-y)
Supplement: Supplementary file 2 — (DOCX 13 kb) [file 10802_2016_224_MOESM2_ESM.docx]

Supplementary Table S2. Effect Sizes for Group Differences on SDQ scores, for step 2 (*Hedge’s* *G*)

| *SDQ scores by age* | EOP vs. Low | Fish  (<2 svgs/wk vs. ≥2 svgs/wk) ^b^ | EOP: Fish  (<2 svgs/wk vs. ≥2 svgs/wk) ^b^ | Low: Fish  (<2 svgs/wk vs. ≥2 svgs/wk) ^b^ |
| --- | --- | --- | --- | --- |
| *4-10 years* |  |  |  |  |
| Emotional Difficulties | 0.97*** | 0.04 | 0.03 | 0.01 |
| Hyperactivity | 1.54*** | 0.06 | 0.10 | 0.01 |
| *12-13 years* |  |  |  |  |
| Emotional Difficulties | 0.99*** | 0.07 | 0.16* | 0.03 |
| Hyperactivity | 1.60*** | 0.06 | 0.03 | 0.03 |
| *SDQ scores by age* | EOP vs. Low | Processed Food  (<1 svg/day vs. ≥1 svg/day) ^b^ | EOP: Processed Food  (<1 svg/day vs. ≥1 svg/day) ^b^ | Low: Processed Food  (<1 svg/day vs. ≥1 svg/day) ^b^ |
| *4-10 years* |  |  |  |  |
| Emotional Difficulties | 0.98*** | 0.08 | 0.07 | 0.07 |
| Hyperactivity | 1.54*** | 0.23** | 0.22** | 0.22** |
| *12-13 years* |  |  |  |  |
| Emotional Difficulties | 0.99*** | 0.14* | 0.38** | 0.09 |
| Hyperactivity | 1.61*** | 0.20** | 0.17* | 0.19* |

*Note*. EOP=early-onset persistent conduct problems; Low CP=low conduct problems; Effect sizes measured as Hedge’s *G* due to difference in sample sizes: *small effect size, **medium effect size, ***large effect size.

^a^ “<2 servings/week” vs. “≥2 servings/week”

^b^ “<1 servings/day” vs. “≥1 servings/day”
